# The protective effects of propofol against CoCl_2_-induced HT22 cell hypoxia injury via PP2A/CAMKIIα/nNOS pathway

**DOI:** 10.1186/s12871-017-0327-1

**Published:** 2017-02-28

**Authors:** Yan Lu, Wei Chen, Chen Lin, Jiaqiang Wang, Minmin Zhu, Jiawei Chen, Changhong Miao

**Affiliations:** 1 Department of Anesthesiology, Fudan University Shanghai Cancer Centre, No. 270 DongAn Road, Shanghai, 200032 People’s Republic of China; 2 Department of Oncology, Shanghai Medical College, Fudan University, Shanghai, 200032 People’s Republic of China; 3 Department of Medical Oncology, Fudan University Shanghai Cancer Centre, No. 270 DongAn Road, Shanghai, 200032 People’s Republic of China

**Keywords:** Propofol, Hypoxia, Cognitive impairment, HT22

## Abstract

**Background:**

Perioperative cerebral ischemia/hypoxia could induce hippocampal injury and has been reported to induce cognitive impairment. In this study, we used cobalt chloride (CoCl_2_) to build a hypoxia model in mouse hippocampal cell lines. Propofol, a widely used intravenous anesthetic agent, has been demonstrated to have neuroprotective effect. Here, we explored whether and how propofol attenuated CoCl_2_-induced mouse hippocampal HT22 cell injury.

**Methods:**

Mouse hippocampal HT22 cells were pretreated with propofol, and then stimulated with CoCl_2_. Cell viability was measured by cell counting kit 8 (CCK8). The effect of propofol on CoCl_2_-modulated expressions of B-cell lymphoma 2 (Bcl-2), BAX, cleaved caspase 3, phosphatase A2 (PP2A), and the phosphorylation of Ca^2+^/Calmodulin (CaM)-dependent protein kinase II (pCAMKIIα), neuron nitric oxide synthase at Ser^1412^ (pnNOS-Ser^1412^), neuron nitric oxide synthase at Ser^847^ (pnNOS-Ser^847^) were detected by Western blot analysis.

**Results:**

Compared with control, CoCl_2_ treatment could significantly decrease cell viability, which could be reversed by propofol. Further, we found CoCl_2_ treatment could up-regulate the expression of PP2A, BAX, cleaved caspase three and cause the phosphorylation of nNOS-Ser^1412^, but it down-regulated the expression of Bcl-2 and the phosphorylation of CAMKIIα and nNOS-Ser^847^. More importantly, these CoCl_2_-mediated effects were attentuated by propofol. In addition, we demonstrated that propofol could exert similar effect to that of the PP2A inhibitor (okadaic acid). Further, the PP2A activator (FTY720) and the CAMKIIα inhibitor (KN93) could reverse the neuroprotective effect of propofol.

**Conclusion:**

Our data indicated that propofol could attenuate CoCl_2_-induced HT22 cells hypoxia injury via PP2A/CAMKIIα/nNOS pathway.

## Background

Postoperative cognitive dysfunction (POCD) is a long-term cognitive impairment after surgery and is becoming one of the most ever-growing concerns in aged patients [1]. Transient global cerebral ischemia/hypoxia is one of the major complications of several clinical situations such as cardiac arrest and severe intraoperative systemic hypotension [2] and has been implicated in the development of POCD [2–6]. Perioperative ischemic/hypoxic brain injury often leads to irreversible brain damage, resulting hippocampal neuron cells injury and was considered the third cause of death and permanent disability [7]. On cellular level, multipline parameters, such as mitochondrial dysfunction and cell apoptosis, have been widely used to represent cell injury. On molecular level, neuron nitric oxide synthase (nNOS) has been reported to be involved in the pathogenesis of cerebral ischemia/hypoxia injury. Activation of nNOS plays a crucial role in neuronal injury after cerebral ischemia/hypoxia [8]. A growing body of evidence suggested that nNOS phosphorylated at Ser^1412^ by phosphatase A2 (PP2A) could be a marker of activation of its enzyme activity [9]. In contrast, Ca^2+^/Calmodulin (CaM)-dependent protein kinase II (CAMKIIα) phosphorylates nNOS at Ser^847^ leading to a reduction of its enzyme activity [10]. In addition, previous study has demonstrated that CAMKIIα could be dephosphorylated by PP2A, leading to a reduction of its enzyme activity [11]. Putting together, inhibition of the activity of PP2A may protect hippocampal cells from injury in vitro.

Propofol, 2,6-diisopropylphenol, has been widely used for the induction and maintenance of general anesthesia in clinical practice. Many studies have indicated its protective effects in multiple organs and tissues, such as cardiovascular system [12], respiratory system [13] and urinary system [14]. In central nervous system, propofol has been demonstrated to be neuroprotective against oxide stress [15] and ischemia injury [16]. However, the underlying mechanism is unclear. In the present study, we used cobalt chloride (CoCl_2_) to build an in vitro hypoxia model and aimed to clarify whether and how propofol attenuated CoCl_2_-induced HT22 cell hypoxia injury.

## Methods

### Cell culture and reagents

HT22 cells were obtained from GuangZhou Jennio Bio- tech and maintained in DMEM (HyClone Laboratories, Logan, Utah, USA) with 5 mM glucose and 10% fetal bovine serum. Cells were incubated in a humidified atmosphere with 5% CO_2_ at 37 °C and sub-cultured when reaching 90% confluence. The eighth passage was used in the present study.

Propofol (Sigma, St. Louis, MO, USA), PP2A inhibitor okadaic acid (Sigma, St. Louis, MO, USA), and PP2A activator FTY720 (Sigma, St. Louis, MO, USA) were dissolved in DMSO (Sigma, St. Louis, MO, USA). In order to avoid possible nonspecific effects, the final concentration of DMSO was adjusted to 0.01% for each solution. A 500 mM stock solution of CoCl_2_ was prepared by dissolving CoCl_2_ powder (Sigma-Aldrich, Dorset, UK) in serum-free DMEM.

### Study design

HT22 cells were treated with CoCl_2_ for 0, 1, 2, 6, 12 and 24 h respectively. By measuring cell viability, we determined the appropriate CoCl_2_ treatment condition with significant effect on cell viability inhibition. During general anesthesia, the concentration of propofol in brain ranges from 4 to 20 μg/ml, which is about 20–100 μM [17]. Therefore, HT22 cells were pretreated with propofol for 2 h with different concentrations (5, 10, 25, 50 μM) to observe its protective effects, and the concentration of maximal protective effects was determined. In the following experiments, the optimal treatment time and concentration of CoCl_2_ and propofol were used to investigate potential mechanisms.

### Analysis for cell viability

Cell viability was maesured by cell counting kit-8 (CCK8) (Dojindo Laboratories, Kumamoto, Japan) according to the manufacture’s instruction. Briefly, 5 × 10^3^ cells per well were plated in 96-well plates and incubated in 37 °C. After designed treatments, 10 μl CCK-8 was added in each well and the 96-well plate was incubated in 37 °C for 2 h. Absorbance at a 450 nm wavelength of each well was determined by a microplate reader (Synergy H4, Bio-Tek). Accounting the mean value and standard deviation of optical density for every six wells was used to draw the cell viability curve.

### Western blot analysis

After corresponding treatment, cells were harvested, washed with cold 1 × PBS, and lysed with RIPA lysis buffer (Beyotime Institute of Biotechnology, Shanghai, China) for 30 min on ice, then centrifuged at 12,000 g for 15 min at 4 °C. The protein concentration was determined by BCA protein assay kit (Beyotime Institute of Biotechnology, Shanghai, China). Equal amount (40 μg) of proteins obtained from different samples were separated by 8 or 10% SDS-PAGE electrophoresis and transferred to polyvinylidene fluoride (PVDF) membranes (Millipore). The PVDF membranes were incubated with primary antibodies at 4 °C overnight after being blocked with 5% skim milk. The primary antibodies used were monoclonal antibody against β-actin (Santa Cruz Biotechnology, Santa Cruz, CA, USA), PP2A (Cell Signaling Technology, Danvers, MA, USA), CAMKIIα (abcam, Cambridge, UK), pCAMKIIα (abcam, Cambridge, UK), nNOS (Santa Cruz Biotechnology, Santa Cruz, CA, USA), pnNOS-Ser^1412^ (abcam, Cambridge, UK), pnNOS-Ser^847^ (abcam, Cambridge, UK), BAX (Cell Signaling Technology, Danvers, MA, USA), Bcl-2 (proteintech, Shanghai, China), caspase 3 (Cell Signaling Technology, Danvers, MA, USA). Thereafter, the PVDF membranes were incubated with secondary antibodies conjugated with horseradish peroxidase (HRP). The protein bands were developed with the chemiluminescent reagents (Millipore, MA, USA). The software of image j was used to analyze the respective densities of the protein bands. In the present study, β-actin was used as loading control and the data were expressed as the ratio of specific protein expression to β-actin expression.

### Statistical analysis

Data were obtained from at least five separately performed experiments and calculated with using Graph Pad Prism. Results were expressed as mean ± SD. An ANOVA was used to determine the levels of significance of differences among various treatments. A value of *p* < 0.05 was considered significant.

## Results

### CoCl_2_ induced HT22 cell injury, which was attenuated by propofol

**Fig. 1:**
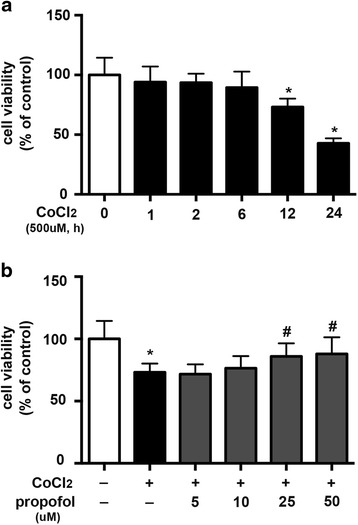
Propofol attenuated CoCl_2_-induced HT22 cell injury. **a** In HT22 cells, 500 μM CoCl_2_ treatment induced cell injury in a time-dependent manner, and 12 h treatment significantly reduced cell viability. **b** 25 μM propofol significantly reduced CoCl_2_-induced cytotoxicity. (* *p* < 0.05 vs. control, # *p* < 0.05 vs. CoCl_2_ treatement, *n* = 5, Data were shown as mean ± SD)

In HT22 cells, 500 μM CoCl_2_ treatment induced cell injury in a time-dependent manner. As shown in Fig. 1a, we found that 500 μM CoCl_2_ treatment for 12 h significantly reduced cell viability by 27% (*p* < 0.05). During general anesthesia, the concentration of propofol in brain ranges from 4 to 20 μg/ml,which is about 20–100 μM [17]. Therefore, HT22 cells were pretreated with propofol for 2 h with different concentrations (5, 10, 25, 50 μM) to observe its protective effects. As shown in Fig. 1b, 25 μM propofol showed a significantly protective effect. Compared with CoCl_2_ treatment, propofol (25 μM, 2 h) restored cell viability by 12% (*p* < 0.05). Thereafter, 12 h treatment of 500 μM CoCl_2_ and 25 μM of propofol pretreatment for 2 h were used in the following experiments to study the signaling pathway involved in the protective effects of propofol.

### CoCl_2_ up-regulated BAX and caspase three expression, and down-regulated Bcl-2 expression, which could be modulated by propofol

**Fig. 2:**
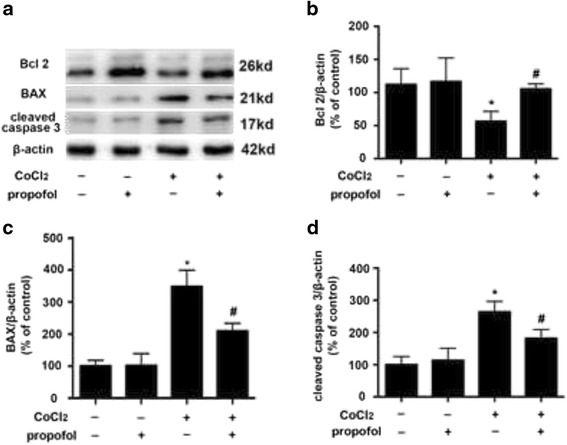
The effects of propofol on CoCl_2_-induced BAX, Bcl-2 and cleaved caspase three expression. **a** CoCl_2_-induced BAX and cleaved caspase 3 overexpression was attenuated by propofol. Bcl2 down-regulation by CoCl_2_ was reversed by propofol. **b**, **c** and **d** Western blot and densitometric quantification of Bcl2, BAX and cleaved caspase three expression. (* *p* < 0.05 vs. control, # *p* < 0.05 vs. CoCl_2_ treatement, *n* = 5, Data were shown as mean ± SD)

Compared with control, CoCl_2_ (500 μM, 12 h) treatment increased the expression of pro-apoptotic protein BAX by 348% (*p* < 0.05, Fig. 2a, c) and the expression of cleaved caspase 3 by 264% (*p* < 0.05, Fig. 2a, d), while it decreased the expression of anti-apoptotic protein Bcl2 by 56% (*p* < 0.05, Fig. 2a, b). However, these effects were reversed by 25 μM propofol treatment (*p* < 0.05, Fig. 2). More importantly, compared with control, propofol treatment alone had no significant effect on the expression of these proteins.

### CoCl_2_ up-regulated PP2A and pnNOS-Ser^1412^ expression, and down-regulated pCAMKIIα and pnNOS-Ser^847^ expression, which could be modulated by propofol

**Fig. 3:**
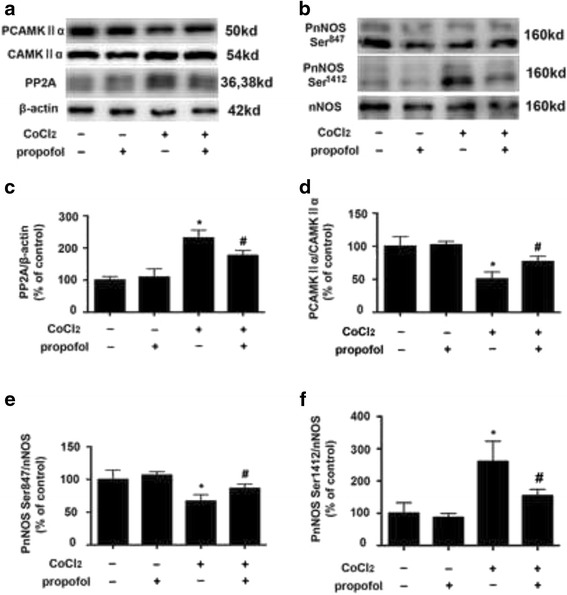
The effects of propofol on CoCl_2_-induced PP2A, pCAMKIIα, pnNOS-Ser^1412^, pnNOS-Ser^847^ expression **a** and **b**, CoCl_2_-induced PP2A and pnNOS-Ser^1412^ overexpression was attenuated by propofol. pCAMKIIα and pnNOS-Ser^847^ down-regulation by CoCl_2_ was reversed by propofol. **c**, **d**, **e** and **f**, Western blot and densitometric quantification of PP2A, pCAMKIIα, pnNOS-Ser^1412^, pnNOS-Ser^847^ expression. (*p < 0.05 vs. control, #*p* < 0.05 vs. CoCl_2_ treatement, *n* = 5, Data were shown as mean ± SD)

Compared with control, CoCl_2_ (500 μM, 12 h) treatment increased the expression of PP2A by 231% (*p* < 0.05, Fig. 3a, c), which was inhibited by 25 μM propofol treatment (*p* < 0.05, Fig. 3a, c).

We also demonstrated that CoCl_2_ (500 μM, 12 h) treatment decreased the expression of pCAMKIIα by 50% (*p* < 0.05, Fig. 3a, d), which was reversed by 25 μM propofol treatment (*p* < 0.05, Fig. 3a, d).

Compared with control, CoCl_2_ (500 μM, 12 h) treatment decreased the expression of pnNOS-Ser^847^ by 67% (*p* < 0.05, Fig. 3a, e) but increased the expression of pnNOS-Ser^1412^ by 261% (*p* < 0.05, Fig. 3a, f), which was reversed by 25 μM propofol treatment (*p* < 0.05, Fig. 3a, e and f).

Similarly, compared with control, propofol treatment alone had no significant effect on the expression and phoshoylation of these proteins.

### CoCl_2_-inhibited cell viability, up-regualted PP2A and pnNOS-Ser^1412^ expression, and down-regulated pCAMKIIα and pnNOS-Ser^847^ expression, which could be modulated by propofol, PP2A inhibitor okadaic acid, PP2A activator FTY720, CAMKIIα inhibitor KN93

To confirm the role of PP2A, we used the PP2A inhibitor (okadaic acid) and the PP2A activator (FTY720). And to confirm the role of *CAMKIIα, we used the CAMKIIα inhibitor (KN93).*

**Fig. 4:**
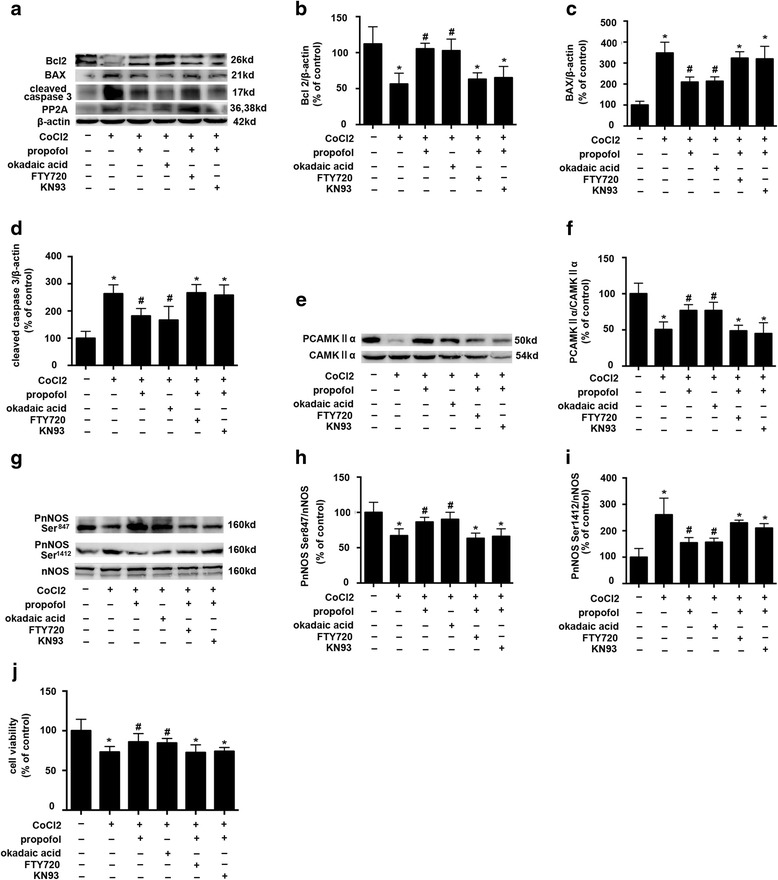
The effects of propfol, okadaic acid, FTY720 and KN93 on CoCl_2_-induced inhibition of cell viability, and the epression of PP2A, pCAMKIIα, pnNOS-Ser^1412^, pnNOS-Ser^847^ expression **a**, **b**, **c** and **d** CoCl_2_-induced BAX, PP2A and cleaved caspase 3 overexpression was attenuated by propofol. Bcl2 down-regulation by CoCl_2_ was reversed by propofol and okadaic acid. Besides PP2A, the effects of propofol could be reversed by FTY720 and KN93. **e** and **f** pCAMKIIα down-regulation by CoCl_2_ was reversed by propofol and okadaic acid. The effects of propofol could be reversed by FTY720 and KN93. **g**, **h** and **i** CoCl_2_-induced pnNOS-Ser^1412^ overexpression was attenuated by propofol and pnNOS-Ser^847^ down-regulation by CoCl_2_ was reversed by propofol and okadaic acid. The effects of propofol could be reversed by FTY720 and KN93. **j** propofol and okadaic acid attenuated CoCl_2_-induced cytotoxicity. The effects of propofol could be reversed by FTY720 and KN93. (* *p* < 0.05 vs. control, # *p* < 0.05 vs. CoCl_2_ treatement, *n* = 5, Data were shown as mean ± SD)

Compared with CoCl_2_ treatment, okadaic acid decreased the expression of PP2A and BAX, but increased the expression of Bcl-2 (*p* < 0.05, Fig. 4a, b and c), which were similar to the effect of propofol. Moreover, FTY720 and KN93 could reverse the effects of propofol. However, the effect of propofol on CoCl_2_-induced PP2A expression was not affected by KN93, which indiated that the phosphorylation of CAMKIIα was modulated by PP2A.

Compared with CoCl_2_ treatment, okadaic acid increased the expression of pCAMKIIα (*p* < 0.05, Fig. 4e, f), which was similar to the effect of propofol treatment. Moreover, FTY720 and KN93 could reverse the effects of propofol.

Compared with CoCl_2_ treatment, okadaic acid decreased the expression of pnNOS-Ser^1412^ but increased the expression pnNOS-Ser^847^ (*p* < 0.05, Fig. 4g, h and i), which was similar to the effect of propofol treatment. Moreover, the effects of propofol could be reversed by FTY720 and KN93.

As shown in Fig. 4j, okadaic acid attenuated CoCl_2_-induced cell injury (87.5 vs 73.1%, *p* < 0.05), which was similar to the effect of propofol treatment. Moreover, FTY720 and KN93 could reverse the effects of propofol.

## Discussion

The major finding of the present study is that in mouse hippocampal HT22 cells, CoCl_2_ activated PP2A, thus inhibiting CAMKIIα phosphorylation and increasing nNOS activity, resulting in increased expression of pro-apoptotic protein BAX and caspase 3 activity. All these effects lead to the inhibition of cell viability. Further, propofol could protect HT22 cells against CoCl_2_-induced apoptosis and cell injury. Our data also suggested that the mechanisms of the protective effects of propofol may involve down-regulating PP2A expression, thus inducing CAMKIIα phosphorylation and inhibiting nNOS activity, resulting in anti-apoptotic protein Bcl-2 expression and therefore reversing cell viability.

Emerging evidence has suggested that ischemia stroke and transient cerebral ischemia/hypoxia promote cognitive impairments in multiple nervous system diseases, such as Alzheimer's disease and POCD [18–20]. During the perioperative period, clinical situations such as cardiac arrest or severe systemic hypotension could lead to transient global cerebral hypoxia and become a risk factor of POCD. In this study, we used CoCl_2_ to build an in vitro hypoxia model. It is a widely used chemical mimic of hypoxia [21]. As shown in Fig. 1, CoCl_2_ treatment (500 μM, 12 h) significantly decreased cell viability.

CAMKIIα is highly expressed in brain and is especially enriched at excitatory synapses and their postsynaptic densities (PSDs). It plays an important role in long-term potentiation (LTP) of excitatory synapse strength and memory formation [22]. Any noxious stimulus, which inhibited CAMKIIα phosphorylation, could impair cognitive function [23] and its phosphorylation at T286 serves as a hallmark feature of CAMKIIα activity.

PP2A worked as an important regulator of mitochondrial shape and function, and a pervious study showed that PP2A could dephosphorylate and decrease the activation of CAMKIIα. So, we hypothesized that activation of PP2A may result in a neurotoxic effect [24, 25]. Consistently, in the present study, we reported that CoCl_2_ could cause cell injury by activating PP2A and thus inhibiting the phosphorylation of CAMKIIα (Figs. 1, 2 and 3). nNOS, the main nitric oxide donor in the brain, is supposed to produce detrimental effects in neurons after cerebral ischemia [26]. Phosphorylation nNOS at Ser^847^ could inhibit the activity of nNOS and exert neuroprotective effect. In addition, after cerebral ischemia, CAMKIIα phosphorylated nNOS at Ser^847^ and attenuated nNOS activity, which could protect neuron cells from ischemic damage [26]. While the phosphorylation of nNOS at Ser^1412^ by PP2A could increase the activity of nNOS, resulting in neuron injury. As shown in Fig. 3, CoCl_2_ treatment could increase the phosphortlation of nNOS at Ser^1412^, but decrease the phosphorylation of nNOS at Ser^847^.

The widely used intravenous anesthetic in clinical settings, propofol, in addition to its sedative-hypnotic property, previous in vitro and in vivo studies indicated that propofol may have protective effects in neuron system [16, 27]. In the present study, we found propofol attenuated CoCl_2_-induced cell injury by reversing the phosphorylation of CAMKIIα (Fig. 3a, d). In contrast, previous study demonstrated that repeated exposure to propofol impairs spatial learning, inhibits LTP and the noxious effect of propofol was related to CAMKIIα [28]. It is noted that the propofol-mediated modulations on the central nervous system may depend on the exposure time of propofol. Studies have demonstrated that in human umbilical vein endothelial cells, propofol exerted protective effect by inhibiting PP2A. Similarly, in the present study, propofol protect mouse hippocampal HT22 cells from CoCl_2_-induced injury by inhibiting the activation of PP2A (Fig. 3a, c). Further, we used a PP2A inhibitor (okadaic acid), and found the protective effect of propofol was similar to that of okadaic acid. Moreover, the protective effect of propofol could be attenuated by a PP2A activator (FTY720) (Fig. 4). These data indicated that the protective effect of propofol was achieved by inhibiting PP2A expression.

There are some limitations in this study. Firstly, pervious study has reported that CAMKIIα is highly sensitive to intracellular Ca^2+^ signaling, and it phosphorylates and up-regulates many of the key proteins involved in intracellular Ca^2+^ loading in ischemia injury [29]. In this study, we only explored the effect of CAMKIIα but did not detecte intracellular Ca^2+^ levels. Secondly, studies have demonstrated that the effects of propofol may be mediated by two different receptors, including gamma-aminobutyric acid (GABA) type receptor [30] and N-methyl-D-aspartate (NMDA) receptor (NR1 and NR2B) [31]. At present, we did not know by which receptor propofol induced the observed effects. Further experiments are required to investigate this issue.

## Conclusion

This study indicated that in mouse hippocampal HT22 cells, CoCl_2_ activated PP2A, thus inhibiting CAMKIIα phosphorylation and increasing nNOS activity, resulting in increased expression of pro-apoptotic protein BAX and caspase three activity. All these effects lead to the inhibition of cell viability. More importantly, we found that propofol could protect HT22 cells against CoCl_2_-induced apoptosis and cell injury. The mechanisms of the protective effects of propofol may involve down-regulating PP2A expression, thus inducing CAMKIIα phosphorylation and inhibiting nNOS activity, resulting in anti-apoptotic protein Bcl-2 expression and therefore reversing cell viability.

## Abbreviations

Bcl-2: B-cell lymphoma 2
CAMKIIα: Ca^2+^/Calmodulin (CaM)-dependent protein kinase IIα
CCK8: Cell counting kit 8
CoCl_2_: Cobalt chloride
GABA: Gamma-aminobutyric acid
HRP: Horseradish peroxidase
LTP: Long-term potentiation
NMDA: N-methyl-D-aspartate
nNOS: Neuron nitric oxide synthase
pCAMKIIα: Phosphorylation of Ca^2+^/Calmodulin (CaM)-dependent protein kinase II
pnNOS-Ser^1412^: Phosphorylation of neuron nitric oxide synthase at Ser^1412^
pnNOS-Ser^847^: Phosphorylation of neuron nitric oxide synthase at Ser^847^
POCD: Postoperative cognitive dysfunction
PP2A: Phosphatase A2
PSDs: Postsynaptic densities
PVDF: Polyvinylidene fluoride
